# Editorial: The COVID-19 pandemic, problematic internet use, post-traumatic stress and mental health

**DOI:** 10.3389/fpsyt.2023.1273778

**Published:** 2023-08-25

**Authors:** Xue Yang, Qian Li, Kylie Kai-yi Chan, Anise M. S. Wu, Miguel Ramos, Xin Wang, Guohua Zhang

**Affiliations:** ^1^Centre for Health Behaviours Research, Jockey Club School of Public Health and Primary Care, Faculty of Medicine, The Chinese University of Hong Kong, Hong Kong, Hong Kong SAR, China; ^2^Faculty of Social Sciences, University of Macau, Macao, China; ^3^Department of Social Policy, Sociology and Criminology, University of Birmingham, Birmingham, United Kingdom; ^4^Department of Psychology, School of Mental Health, Wenzhou Medical University, Wenzhou, China

**Keywords:** COVID-19, pandemic, internet use, post-traumatic stress, mental health

The COVID-19 pandemic has caused significant loss of life and disruptions to daily activities worldwide, which can be traumatic and have short-term and long-term effects on mental health. Although public health measures such as social distancing, quarantine, and lockdowns can facilitate disease control, they may exacerbate feelings of fear, worry, stress, and social isolation, leading to mental health issues. While the internet has become increasingly important in coping with pandemic-related stress, excessive reliance on it may lead to problematic internet use and associated health risks. This Research Topic curated a collection of papers that are representative of current trends and advances in discussing and investigating the complex relationships among internet use, COVID-19 related stress and mental health issues.

The Research Topic has shown that COVID-19 has had a significant impact on mental health in different populations worldwide. For example, in Italy, 20–30% of ICU patients infected with COVID-19 exhibited depression or anxiety and perceived stress at 1 month and 6 months after discharge from hospital. At 6 months, 7% of patients showed post-traumatic stress symptoms (Carola et al.). In China, adults experiencing self-infection or close contact reported the highest levels of distress compared to those who experienced neighborhood infection or family member infection (Xiong et al.). COVID-19 survivors also reported stress related to stigma and discrimination in intimate social relationships, workplaces, and children's schools, with sexual minorities who were involved in mass disease transmission experiencing a higher level of stigmatization (Kang et al.). The impact of COVID-19 on mental health was not limited to those who were infected, but also extended to the general public and medical professionals. Over 20% of college students reported depressive symptoms during the pandemic (Luo et al.). In addition, Liu et al. investigated the sleep quality, fatigue, and mental workload and their contributing factors among clinical first-line nurses working in three tertiary hospitals. It was reported that 49% of nurses' mental workload was severely affected by COVID-19, and 68.9% of nurses' sleep quality was slightly affected (Liu et al.). Addressing these COVID-19 related mental health and stigma issues is crucial as such knowledge will help to prevent related issues in future emerging pandemics.

Furthermore, internet use during the pandemic has played a mixed role in mental health. On one hand, internet use caused a series of mental health issues and behavior problems. For example, Zhang et al. found that attachment anxiety affected smartphone addiction among university students during the COVID-19 confinement, with teacher-student relationships and school connectedness mediating the relationship. Among school teachers, problematic gaming had stronger negative influence than problematic social media use on their psychological distress during online teaching due to COVID-19 (Chen et al.). On the other hand, several studies reported a protective effect of internet use during the COVID-19 pandemic. Teng found mobile internet use significantly alleviated mental distress in Chinese adults through trust and happiness. Huang et al. reported that the online risk information ground factors significantly affect online users' perceptions of health risks, and trust in official media was linked to reduced depressive symptoms. They highlighted the importance of fostering public trust in official media through rapid dissemination and transparency of information in mitigating the negative impact of COVID-19 (Huang et al.).

Future research directions on this topic should focus on several key areas. First, more intervention studies are needed to identify effective strategies to cope with mental health, behavioral health, and stigma issues during and after pandemics. The COVID-19 pandemic has highlighted the need for innovative and effective interventions (e.g., digital health techniques, transdiagnostic interventions) to support mental health in the unusual circumstances such as quarantine and lockdowns, which have challenged the applicability of conventional interventions. Research in this area can help identify strategies that are most effective in the context of pandemics. Second, more longitudinal studies are needed to establish the causal relationship between internet use and mental health outcomes given the mixed findings from existing cross-sectional studies. Third, social and health minority groups deserve particular attention as the pandemic may have added extra burdens to their life (e.g., intersectional stigma, health equity). Last but not least, there is a need to better understand the underlying mechanisms that link internet use and mental health outcomes and the reason for its mixed role. This may involve exploring factors such as the purpose of internet use, the source and the reliability of online information. Since internet use has become an indispensable part of everyday life, research in this area is important to delineate the relationship between internet use on mental health outcomes and the potential mechanisms in order to inform best practices on education and training, health promotion and early identification of internet users of at-risk mental health.

## Author contributions

XY: Writing—review and editing. QL: Writing—original draft. KC: Writing—review and editing. AMSW: Writing—review and editing. MR: Writing—review and editing. XW: Writing—review and editing. GZ: Writing—review and editing.

